# Investigating the role of the metabolic score for visceral Fat in assessing the prevalence of chronic kidney disease from the NHANES 1999–2018

**DOI:** 10.1038/s41598-025-86723-3

**Published:** 2025-01-18

**Authors:** Xingcheng Zhou, Jiayi Xiang, Shuxian Zhang, Jun Yang, Yi Tang, Yalan Wang

**Affiliations:** 1 Department of Pathology, College of Basic Medicine, Chongqing Medical University, Chongqing, China; 2 Molecular Medicine Diagnostic and Testing Center, Chongqing Medical University, Chongqing, China; 3 Department of Clinical Pathololgy Laboratory of Pathology Diagnostic Center, Chongqing Medical University, Chongqing, China; 4 Fifth Department of Medicine (Nephrology/Endocrinology/Rheumatology), University Medical Centre Mannheim, University of Heidelberg, Mannheim, Germany; 5 Department of Anesthesiology, The First Affiliated Hospital of Chongqing Medical University, Chongqing, China

**Keywords:** Computational biology and bioinformatics, Endocrinology, Nephrology, Kidney diseases, Public health

## Abstract

This study investigates the association between the Metabolic Score for Visceral Fat (METS-VF) and chronic kidney disease (CKD), assessing METS-VF as a potential predictor of CKD risk. Utilizing data from the 1999–2018 National Health and Nutrition Examination Survey (NHANES), this cross-sectional study included 24,387 adult participants. Multivariable logistic regression, restricted cubic spline models, and threshold effect analyses were employed to explore the relationship. The results revealed a significant positive association, with multivariable logistic regression showing that each unit increase in METS-VF was associated with an 86% higher risk of CKD (OR: 1.86, 95% CI: 1.48–2.34). Critical METS-VF thresholds (6.10 and 7.55) were identified, at which CKD risk increased substantially. Subgroup analyses indicated that the association was particularly pronounced among older adults and males. These findings suggest that METS-VF is a reliable predictor for assessing CKD risk and that lifestyle interventions, including dietary modifications and exercise programs, may mitigate this risk.

**Supplementary Information:**

The online version contains supplementary material available at 10.1038/s41598-025-86723-3.

## Introduction

Chronic kidney disease (CKD) is defined by a decline in glomerular filtration rate (GFR) or the presence of elevated protein concentrations in the urine. It is a persistent condition with an adult global prevalence of approximately 10%, of which 2% progress to end-stage renal disease (ESRD). In the early stages, CKD often presents without noticeable symptoms, complicating early diagnosis. By the time symptoms manifest, kidney damage is frequently irreversible. If not appropriately managed, CKD may advance to ESRD, requiring life-sustaining interventions, such as hemodialysis or kidney transplantation^1,2^. This progression imposes a considerable psychological burden on patients and generates substantial economic costs. Epidemiological data indicate that the prevalence of CKD among the adult population in the United States varies by age group, with approximately 15.9% in those aged 20–39, 29.0% in those aged 40–59, and 55.1% in those aged ≥ 60^3^.

CKD results from the complex interaction of multiple risk factors, including aging, obesity, genetic predisposition, and environmental influences. Among these, obesity has emerged as a significant, independent risk factor for CKD^4,5^. Body mass index (BMI) is commonly employed to assess an individual’s weight category and associated health risks. However, while elevated BMI is widely recognized as an indicator of increased CKD risk, it predominantly reflects peripheral obesity and fails to adequately account for visceral fat, which is more strongly correlated with metabolic disorders and CKD. Research indicates that individuals with excess visceral fat, even those with a normal BMI, face a markedly higher risk of cardiovascular diseases and metabolic complications^6^. To assess abdominal obesity measures such as waist circumference (WC), hip circumference (HC), and waist-to-hip ratio (WHR) are frequently used. However, these single-measurement indicators cannot effectively differentiate between subcutaneous fat and visceral fat. Although MRI and air displacement plethysmography are considered the gold standards for visceral fat assessment^7,8^, their high costs limit their widespread application, thus necessitating further exploration of the relationship between visceral fat and CKD.

The Metabolism Score for Visceral Fat (METS-VF) is an innovative metric designed to assess visceral adipose tissue (VAT). It is derived from a combination of factors, including age, gender, waist-to-height ratio (WHtR), and the Metabolic Score for Insulin Resistance (METS-IR). This metric has been validated through bioelectrical impedance analysis (BIA) and MRI^9^. While the association between METS-VF and CKD has been demonstrated in Asian populations^10^, no studies have yet investigated this relationship in U.S. populations. Moreover, existing studies are often limited by small sample sizes, a focus on older age groups, and a lack of geographic and population diversity, which may hinder the accurate generalization of findings. To address these limitations, this study presents a novel cross-sectional analysis using NHANES data, incorporating a wide range of variables and reference factors. This approach enhances the robustness of the findings and provides a more comprehensive understanding of relevant risk factors, thereby broadening the scope and improving the representativeness of previous research.

## Materials and methods

### Data sources

The data utilized in this study were sourced from the National Health and Nutrition Examination Survey (NHANES), which is administered by the National Center for Health Statistics (NCHS), a division of the Centers for Disease Control and Prevention (CDC) in the United States. NHANES offers a thorough assessment of the health and nutritional status of the U.S. population. To improve sample representativeness and ensure the accuracy of the findings, a rigorous multi-stage probability sampling method was employed.

**Fig. 1:**
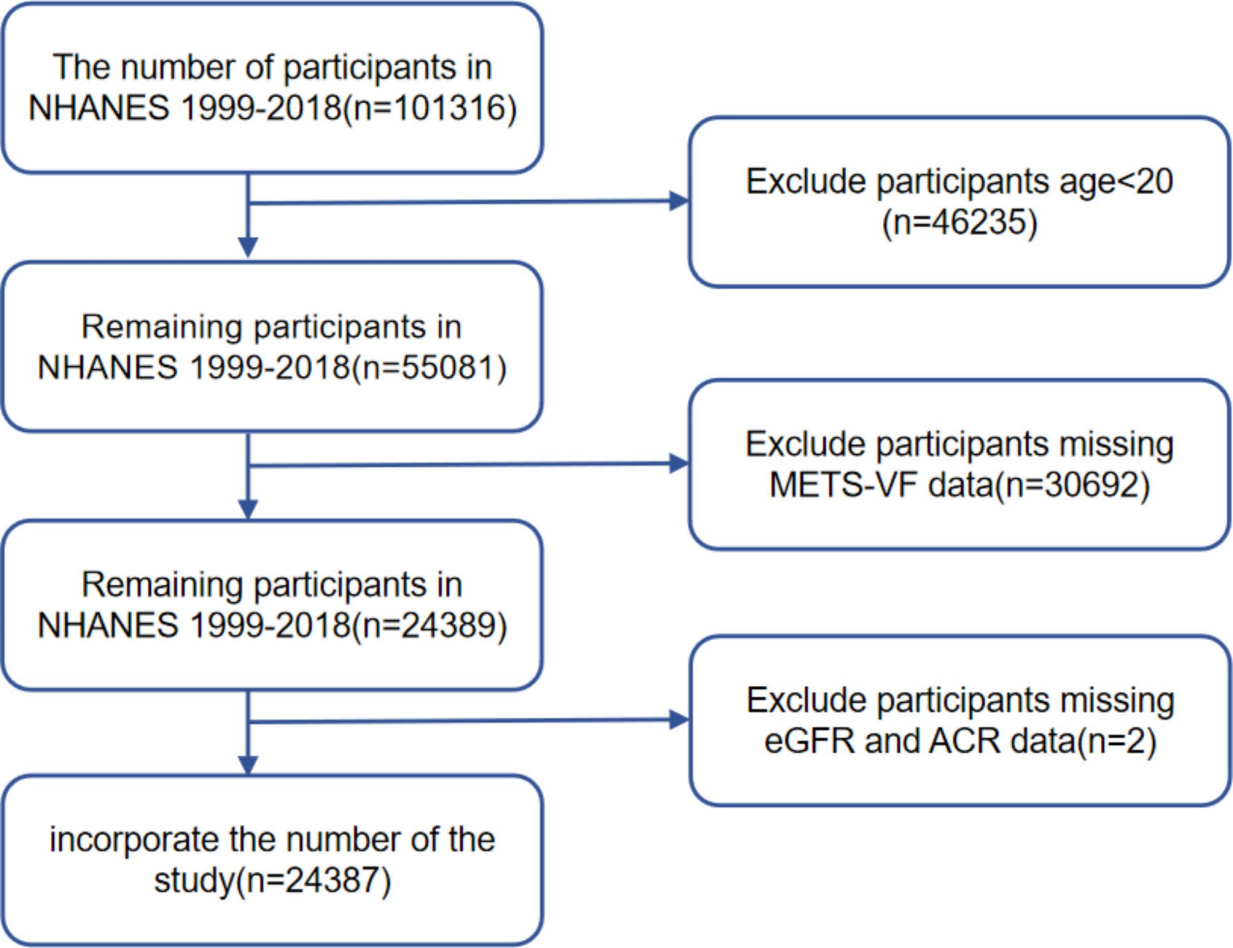
Flow chart for participants.

The study included 101,316 participants from 10 independent NHANES survey cycles between 1999 and 2018. Researchers implemented exclusion criteria for the following groups: participants younger than 20 (*n* = 46,235) were excluded. Additionally, individuals without METS-VF data were excluded (*n* = 30692), as well as subjects with missing renal function data (*n* = 2). After excluding participants with missing data for the aforementioned variables, this cross-sectional study included 24,387 participants for further analysis (Fig. 1).

## Definition of METS-VF and CKD

(1)In this study, METS-VF served as the main exposure factor to evaluate the level of visceral fat accumulation and the metabolic health status of the participants.

METS-VF is calculated by integrating multiple metabolic-related indicators, including WHtR, METS-IR, gender, and age. The calculation formula is as follows:

$$\begin{aligned}{\rm METS-VF}=4.466 + 0.011 \times ({\rm Ln(METS-IR))^{3}}+3.329\times (\rm Ln(WHtr))^{3}\:\\+0.319 \times({\rm gender}) + 0.594 \times ((\rm Ln(Age))\end{aligned}$$

$$\:{\rm METS-IR}=\frac{{\rm Ln((2+FPG)+TG})\times{\rm BMI}}{{\rm Ln(HDF-C)}}$$

$${\rm WHtr}=\frac{{\rm Waist\:Circumference(cm)}}{\rm Hight(cm)}$$

· Gender is coded as a binary variable (1 for males, 0 for females), Age is measured in years, Fasting Plasma Glucose (FPG) in mg/dL, triglycerides (TG) in mg/dL, BMI in kg/m², and High-density lipoprotein cholesterol (HDL-C) in mg/dL.

(2)LAP(lipid accumulation product).

$${\rm LAP=(WC(cm)-(Gender))\times (TG(mmol/L))}$$

· Gender here is a sex specific correction constant (65 for males, 58 for females).

(3)VAI(Visceral Adiposity Index).

$$\begin{aligned}{\rm VAI(men)=\frac{WC(cm)}{39.68+1.88\times {\rm BMI}}}\times \frac{{\rm TG(mmol/L))}}{1.03}\times \frac{1.31}{{\rm HDL-C(mmol/L)}}\:\\ \:{\rm VAI(Women)}=\frac{{\rm WC(cm)}}{36.58+1.89\times{\rm BMI}}\times\frac{{\rm TG(mmol/L)}}{0.81}\times\frac{1.52}{{\rm HDL-C(mmol/L})}\end{aligned}$$

(4)BRI(Body Roundness Index).

$${\rm BRI}=364.2-365.5\sqrt{1-\frac{(\frac{{\rm WC(cm)}}{2\pi}^{2}}{(0.5\times{\rm Hight(cm)}^{2}}}$$

According to the review of the literature and epidemiological guidelines, the criteria for evaluating CKD are as follows: (1)Estimated glomerular filtration rate(eGFR) < 60 ml/min/1.73 m^2^, (2)Urinary albumin/creatinine ratio(UACR) > 30 mg/g. The calculation formula is as follows:

$${\rm eGFR=141*min(\frac{Scr}{K},1)^{\alpha}}*\:{\rm max(\frac{Scr}{K},1)^{-1.209}*0.993^{Age}*}$$

$$1.018^{\rm if \:female}*1.159^{\rm if \:black}$$

It is essential to highlight that in the aforementioned formula, Scr represents the concentration of blood creatinine measured in milligrams per deciliter (mg/dL). The variable K is assigned a value of 0.9 for males and 0.7 for females, whereas α is designated as −0.411 for males and − 0.329 for females.

$${\rm UACR}=\frac{{\rm Urine\:Albumin\:(mg/dL)}}{\rm Urine\: Creatinine(g/dL)}$$

## Covariates

Besides the primary exposure factor, METS-VF, and the outcome variable, CKD, this study included several potential confounders from the NHANES database to conduct a more comprehensive analysis. These covariates include gender, age, race, education level, the poverty income ratio (PIR), alcohol intake, physical activity, hypertension, diabetes, serum creatinine, serum albumin, low-density lipoprotein cholesterol (LDL-C), high-density lipoprotein cholesterol (HDL-C), triglycerides (TG), urine creatinine, and urine albumin.

The selection of these covariates was informed by the existing literature^11,12^. Specifically, age, gender, hypertension, and diabetes have been well-documented in previous studies as significant determinants of CKD prevalence. Additionally, biomarkers such as serum creatinine and albumin, which are routinely used in clinical practice to assess kidney function, were included. Variables influencing metabolic risk factors, such as LDL-C, HDL-C, and triglycerides, as well as lifestyle factors like alcohol intake and physical activity.

Alcohol intake was defined as the intake of at least 12 alcoholic beverages within the past 12 months, and physical activity was defined as engaging in two or more moderate-to-vigorous intensity exercises during the past week or having a self-reported exercise plan documented in the questionnaire. The inclusion of these specific lifestyle and metabolic variables was also supported by their documented relevance in the literature as potential confounders in the relationship between METS-VF and CKD.

### Statistical analysis

To appropriately account for the complex sampling design and ensure that the analysis results are nationally representative, all statistical analyses were adjusted for the survey design and weighted variables. Continuous variables that follow a normal distribution are presented as means with standard deviations (SD), while categorical variables are represented as frequencies and percentages. Logistic regression was used for continuous variables, and the Chi-square test was applied to dichotomous variables.

To enhance the credibility of the results, a multivariable logistic regression analysis was conducted to examine the association between METS-VF quartiles and the prevalence of CKD. Three distinct models were developed: Model 1 was unadjusted, Model 2 adjusted for key demographic factors to control for potential biases, and Model 3 included all covariates from Model 2, as well as additional factors to further reduce confounding and improve result accuracy. Furthermore, a restricted cubic spline (RCS) model was used to analyze the dose-response relationship and identify any nonlinear associations. The likelihood ratio test was applied to assess the nonlinear trend, with nodes positioned at the 25th, 50th, and 75th percentiles of the METS-VF distribution to ensure the robustness and reliability of the findings. Finally, the predictive effects of METS-VF and other indices on CKD were assessed by the receiver operating characteristic (ROC) curve and the area under the curve (AUC).

All data analyses were performed using EmpowerStats Software and R (version 4.4.1) to ensure reproducibility and scientific rigor. Detailed information on the treatment of continuous and categorical variables used in the study is available on the official NHANES website, ensuring transparency and data traceability.

## Results

### Participant characteristics

A total of 24,387 participants from the NHANES dataset were included in this study, with a mean age of 46.03 years and 49.01% male. Of these, 4,055 were diagnosed with CKD. Compared to the non-CKD group, CKD patients were significantly older, with a mean age of 59.60 years, had a higher proportion of females (57.32%), and exhibited an increased average BMI of 29.78 kg/m². Additionally, CKD patients had lower educational levels and a lower Poverty Income Ratio (PIR). The prevalence of hypertension was significantly higher in the CKD group (44.38%), as was the prevalence of diabetes (23.96%). Furthermore, CKD patients engaged in less physical activity and had significantly higher metabolic equivalent (METS-VF) scores (7.66) compared to non-CKD participants (7.28). These findings highlight notable lifestyle and health status differences between CKD and non-CKD populations (Table 1).

**Table 1:** Baselines characteristics of participants(NHANES 1999–2018), weighted.

Variable	Overall	No CKD	With CKD	*P*-value
Age(years)	46.03(17.19)	43.98(16.06)	59.60(18.25)	< 0.001
BMI	28.57(6.63)	28.38(6.48)	29.78(7.38)	< 0.001
Gender				< 0.001
- Male	49.01 (48.11, 49.91)	49.96 (48.98, 50.94)	42.68 (40.46, 44.91)	
- Female	50.99 (50.12, 51.87)	50.04 (49.07, 51.00)	57.32 (55.23, 59.41)	
Race				0.003
- Mexican American	8.37 (7.56, 9.17)	8.47 (7.60, 9.34)	7.67 (5.65, 9.69)	
- Non-Hispanic White	10.61 (9.75, 11.46)	10.38 (9.45, 11.31)	12.11 (9.93, 14.28)	
- Non-Hispanic Black	68.54 (67.66, 69.42)	68.47 (67.49, 69.45)	69.00 (66.95, 71.05)	
- Other Hispanic	5.66 (4.66, 6.66)	5.79 (4.70, 6.88)	4.79 (2.28, 7.29)	
- Other Race	6.83 (5.78, 7.88)	6.89 (5.75, 8.02)	6.44 (3.67, 9.21)	
Education				< 0.001
- Below high school	16.91 (15.97, 17.85)	15.90 (14.86, 16.93)	23.65 (21.39, 25.92)	
- High scool	23.19 (22.05, 24.34)	22.63 (21.37, 23.89)	26.90 (24.11, 29.70)	
- Above high school	59.89 (59.05, 60.73)	61.47 (60.58, 62.37)	49.44 (47.09, 51.80)	
Poverty income ratio (%)				< 0.001
- <1.3	19.84 (18.91, 20.77)	19.35 (18.33, 20.37)	23.10 (20.75, 25.45)	
− 1.3–3.5	40.90 (39.96, 41.83)	39.94 (38.91, 40.97)	47.24 (45.01, 49.46)	
- >3.5	39.26 (38.10, 40.43)	40.71 (39.46, 41.97)	29.67 (26.64, 32.69)	
Alcohol Intake (%)				< 0.001
- No	24.74 (23.78, 25.69)	23.52 (22.48, 24.57)	32.77 (30.38, 35.15)	
- Yes	75.26 (74.61, 75.92)	76.48 (75.77, 77.18)	67.23 (65.42, 69.05)	
Hypertension(%)				< 0.001
- No	76.36 (75.74, 76.98)	79.49 (78.86, 80.12)	55.62 (53.51, 57.73)	
- Yes	23.64 (22.60, 24.68)	20.51 (19.32, 21.69)	44.38 (42.16, 46.60)	
Diabetes(%)				< 0.001
- No	91.73 (91.37, 92.10)	94.10 (93.77, 94.44)	76.04 (74.49, 77.59)	
- Yes	8.27 (7.22, 9.31)	5.90 (4.72, 7.08)	23.96 (21.48, 26.44)	
Exercise Routine(%)				< 0.001
- No	88.72 (88.30, 89.14)	87.64 (87.16, 88.12)	95.88 (95.26, 96.51)	
- Yes	11.28 (10.04, 12.52)	12.36 (11.04, 13.69)	4.12 (0.81, 7.42)	
Serum Albumin(g/dL)	4.25 (0.35)	4.270(0.340)	4.151(0.364)	< 0.001
Serum Creatinine (mg/dL)	0.87(0.35)	0.83(0.18)	1.10(0.80)	< 0.001
Urinary Albumin (ug/mL)	33.29(273.02)	9.53(9.47)	190.60(734.11)	< 0.001
Urinary Creatinine (mg/dL)	129.78(78.44)	131.85(79.35)	116.12(70.64)	< 0.001
Serum triglycerides(mg/dL)	130.49(120.41)	127.01(115.63	153.54(146.15)	< 0.001
Serum LDL(mg/dL)	115.03(34.79)	115.59(34.30)	111.30(37.65)	< 0.001
Serum HDL cholesterol (mg/dL)	53.70(16.15)	53.69(15.78)	53.72(18.44)	0.943
VAI	3.40 (4.50)	3.28(4.22)	4.28(5.89)	< 0.001
LAP	57.46(66.42)	55.44(63.63)	77.28(85.85)	< 0.001
BRI	5.29(2.28)	5.05(2.19)	6.06(2.51)	< 0.001
METS-VF	7.40 (0.80)	7.28(0.76)	7.66(0.72)	< 0.001

The data are summarized as the mean ± SD or median (interquartile range) for continuous variables or as a numerical proportion for categorical variables.

CKD chronic kidney disease, BMI body mass index, HDL high-density lipoprotein, LDL low-density lipoprotein, METS-VF, metabolic score for visceral fat; BRI, body roundness index; LAP, lipid accumulation product; VAI, visceral adiposity index; BMI, body mass index.

## Association between METS-VF and CKD

The findings of this study demonstrate a correlation between elevated METS-VF levels and an increased likelihood of CKD prevalence (Table 2). Model 1 was unadjusted, while Model 2 included adjustments for age, gender, and ethnicity. Model 3 incorporated all covariates from Model 2. A statistically significant association was identified, with an Odds Ratio (OR) of 1.860 (95% Confidence Interval [CI]: 1.480, 2.338), indicating that each unit increase in METS-VF is associated with an 86% increase in the risk of CKD. Upon stratifying participants by METS-VF quartiles, the results remained consistent. Compared to the first quartile (Q1), the positive association remained statistically significant after adjusting for all covariates, with OR (95% CI) values as follows: Q2 = 1.613 (1.239–2.101), Q3 = 1.667 (1.230–2.259), Q4 = 2.259 (1.561–3.268) (Table 2).

**Table 2:** Logistic regression analysis between METS-VF index with CKD prevalence.

OR^1^(95%CI^2^)			
	Model 1	Model 2	Model 3
METS-VF Index	3.328 (3.102, 3.570)	1.743 (1.601, 1.897)	1.860 (1.480, 2.338)
Quartiles of METS-VF			
Q1	Ref.	Ref.	Ref.
Q2	1.493 (1.312, 1.699)	1.098 (0.958, 1.258)	1.613 (1.239, 2.101)
Q3	2.698 (2.394, 3.040)	1.496 (1.305, 1.715)	1.667 (1.230, 2.259)
Q4	6.512 (5.821, 7.286)	2.475 (2.147, 2.853)	2.259 (1.561, 3.268)
*P* for trend	< 0.01	< 0.01	< 0.01

Model 1: no covariate were adjusted.

Model 2: adjusted for age, gender and race.

Model 3: adjusted for age, gender, race, education level, poverty-to-income ratio, alcohol intake, Hypertension, Diabetes, exercise routine, serum albumin, serum creatinine, low-density lipoprotein cholesterol, high-density lipoprotein cholesterol, triglyceride, urinary albumin and urinary creatinine.

**Fig. 2:**
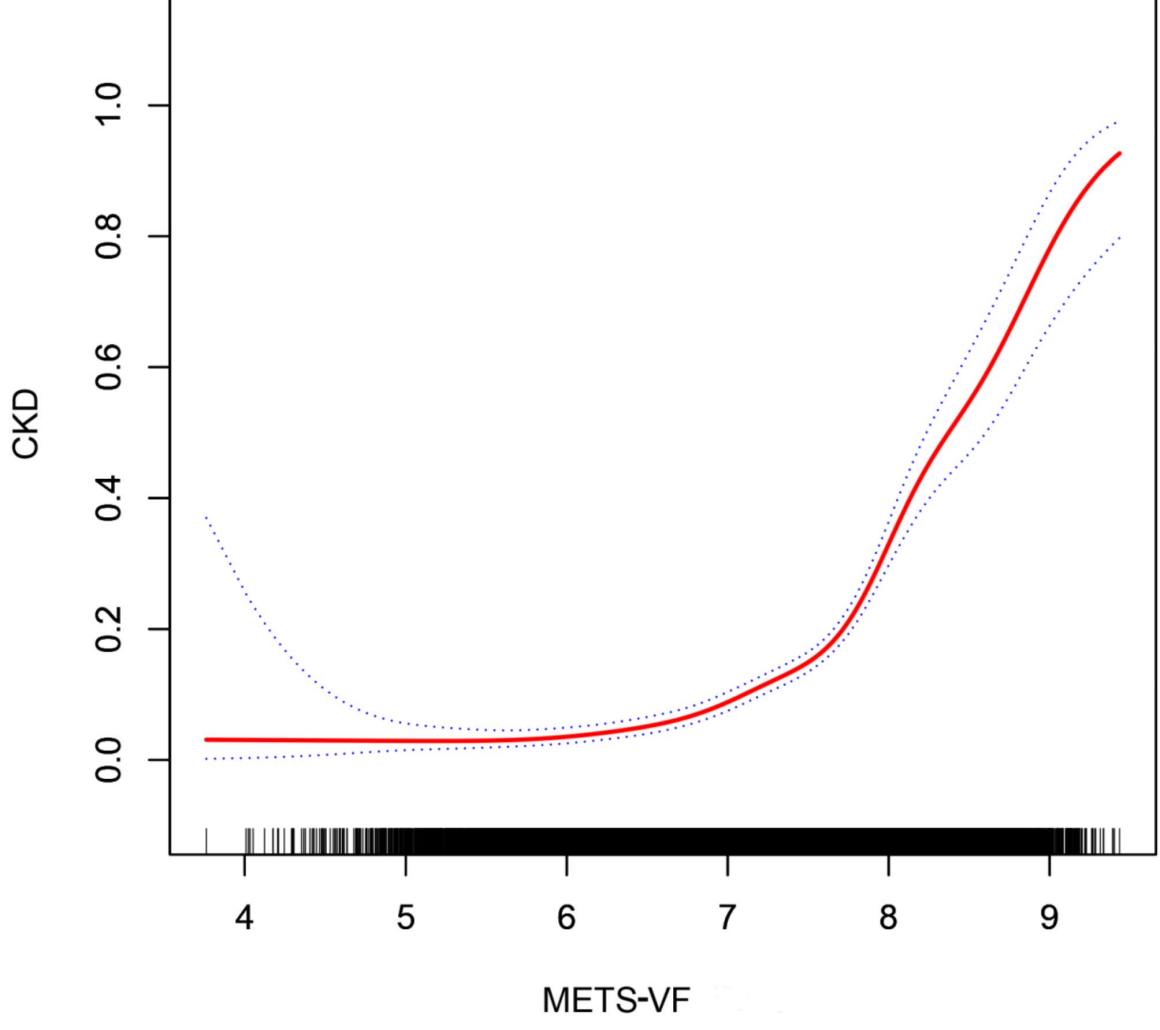
Analysis of the relationship between METS-VF and CKD by smoothing curve fitting The red solid bars indicate a smooth curve fit between METS-VF and CKD, and the blue dashed strip indicates the 95% confidence interval of the fit between METS-VF and CKD. Adjusting variables: age, gender, race, education level, poverty-to-income ratio, alcohol intake, Hypertension, Diabetes, exercise routine, serum albumin, serum creatinine, low-density lipoprotein cholesterol, high-density lipoprotein cholesterol, triglyceride, urinary albumin and urinary creatinine. METS-VF: Metabolism score for visceral fat.

The results from the smooth curve fitting analysis revealed a piecewise between METS-VF levels and CKD prevalence (Fig. 2). Threshold effect analysis identified two critical inflection points at METS-VF levels of 6.10 and 7.55 (Table 3), beyond which the risk of CKD increased substantially. When METS-VF was below 6.10, the odds ratio (OR) for CKD prevalence was 0.84 (95% CI: 0.49–1.44), with no statistically significant association (*p* = 0.53). However, as METS-VF exceeded 6.10, the OR increased significantly to 1.77 (95% CI: 1.58–1.99, *p* < 0.01), indicating a positive correlation between METS-VF and CKD prevalence. Beyond the second threshold of 7.55, the OR surged to 6.61 (95% CI: 2.95–4.74, *p* < 0.01), suggesting a sharp increase in CKD risk. These findings highlight the complex, threshold-dependent relationship between METS-VF and CKD, emphasizing the importance of identifying critical METS-VF levels for early CKD risk stratification.

**Table 3:** The results of the two-piecewise linear regression model.

Outcome	CKD		*P* value
	**β**	(95%CI)	
**Model I**			
A straight-line effect	2.33	(2.08, 2.61)	< 0.01
**Model II**			
Fold points (K1, K2)	6.10, 7.55		
< K1 segment effect 1	0.84	(0.49–1.44)	0.53
K1-K2 segment effect 2	1.77	(1.58–1.99)	< 0.01
> K2 segment effect 3	6.61	(2.95–4.74)	< 0.01
Log likelihood ratio tests	< 0.01		

Adjusted for age, gender, race, education level, poverty-to-income ratio, alcohol intake, Hypertension, Diabetes, exercise routine, serum albumin, serum creatinine, low-density lipoprotein cholesterol, high-density lipoprotein cholesterol, triglyceride, urinary albumin and urinary creatinine.

## Subgroup analysis

**Fig. 3:**
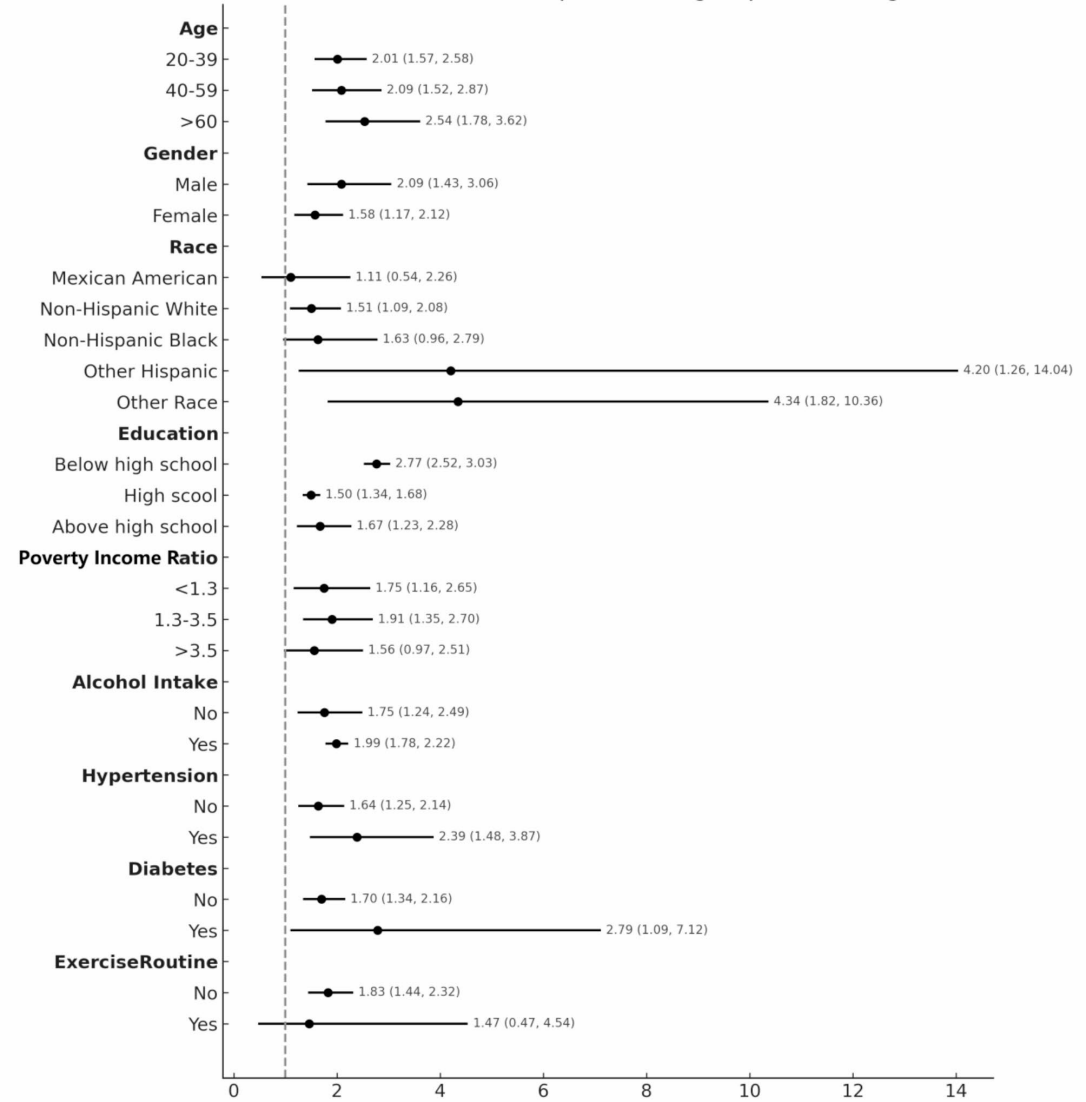
Subgroup analysis of the association between METS-VF and CKD. In the subgroup analysis stratified by each covariate, the model is not adjusted for the stratification variable itself.

Subgroup analysis (Table 4) revealed that elevated METS-VF consistently serves as a risk factor for increased CKD prevalence across diverse populations. The association was particularly significant in the following subgroups: age > 60 years (OR: 2.536 [95% CI: 1.78–3.62]), male (OR: 2.089 [95% CI: 1.43–3.06]), other race (OR: 4.34 [95% CI: 1.82–10.36]), education level below high school (OR: 2.766 [95% CI: 2.53–3.03]), PIR 1.3–3.5 (OR: 1.91 [95% CI: 1.35–2.70]), alcohol intake (OR: 1.99 [95% CI: 1.78–2.22]), hypertension (OR: 2.39 [95% CI: 1.48–3.87]), diabetes (OR: 2.79 [95% CI: 1.10–7.12]), and no exercise routine (OR: 1.83 [95% CI: 1.44–2.32]). These results are illustrated in a forest plot (Fig. 3).

**Table 4:** Subgroup analysis between METS-VF index with CKD prevalence.

Variables	Model 1OR (95%CI)	Model 2OR (95%CI)	Model 3OR (95%CI)
**Age**			
20–39	1.209 (1.106, 1.322)	1.253 (1.147, 1.369)	2.009 (1.567, 2.576)
40–59	2.106 (1.869, 2.373)	1.565 (1.381, 1.774)	2.087 (1.520, 2.865)
> 60	2.892 (2.560, 3.268)	2.053 (1.797, 2.345)	2.536 (1.779, 3.616)
**BMI**			
< 25	2.938 (2.638, 3.272)	1.396 (1.194, 1.633)	1.618 (1.202, 2.179)
25–30	11.329 (9.381, 13.682)	7.714 (5.563, 10.698)	2.602 (1.490, 4.541)
> 30	12.844 (10.526, 15.672)	13.485 (9.191, 19.784)	2.943 (1.395, 6.210)
**Gender**			
Male	5.142 (4.561, 5.795)	2.298 (2.004, 2.634)	2.089 (1.427, 3.058)
Female	2.847 (2.586, 3.134)	1.416 (1.269, 1.581)	1.577 (1.174, 2.119)
**Race**			
- Mexican American	3.598 (2.979, 4.346)	2.013 (1.576, 2.571)	1.106 (0.540, 2.263)
- Non-Hispanic White	3.530 (3.183, 3.916)	1.675 (1.473, 1.904)	1.507 (1.092, 2.078)
- Non-Hispanic Black	2.762 (2.407, 3.168)	1.546 (1.319, 1.812)	1.633 (0.956, 2.789)
- Other Hispanic	3.359 (2.519, 4.479)	2.040 (1.402, 2.969)	4.204 (1.259, 14.039)
- Other Race	3.635 (2.839, 4.654)	2.203 (1.647, 2.945)	4.343 (1.820, 10.362)
**Education**			
- Below high school	4.060 (3.487, 4.728)	3.556 (3.028, 4.176)	2.766 (2.523, 3.033)
- High scool	2.151 (1.803, 2.567)	1.830 (1.513, 2.215)	1.499 (1.338, 1.680)
- Above high school	1.876 (1.175, 2.996)	2.146 (1.235, 3.727)	1.674 (1.227, 2.284)
**PIR**			
< 1.3	2.925 (2.595, 3.296)	1.443 (1.251, 1.665)	1.751 (1.158, 2.649)
1.3–3.5	3.433 (3.092, 3.811)	1.710 (1.507, 1.940)	1.906 (1.346, 2.698)
> 3.5	3.767 (3.225, 4.399)	2.154 (1.778, 2.610)	1.562 (0.973, 2.508)
**Alcohol Intake**			
- No	3.040 (2.730, 3.384)	1.405 (1.229, 1.605)	1.755 (1.237, 2.491)
- Yes	3.686 (3.360, 4.045)	1.990 (1.781, 2.223)	1.990 (1.781, 2.223)
**Hypertension**			
- No	2.627 (2.415, 2.856)	1.479 (1.338, 1.635)	1.638 (1.253, 2.141)
- Yes	2.741 (2.371, 3.169)	1.787 (1.513, 2.111)	2.390 (1.475, 3.873)
**Diabetes**			
- No	2.623 (2.434, 2.826)	1.421 (1.299, 1.554)	1.703 (1.340, 2.163)
- Yes	2.951 (2.327, 3.742)	2.124 (1.610, 2.803)	2.791 (1.095, 7.115)
**Exercise Routine**			
- No	3.301 (3.064, 3.557)	1.780 (1.627, 1.947)	1.827 (1.438, 2.319)
- Yes	1.358 (1.060, 1.739)	1.183 (0.896, 1.562)	1.465 (0.473, 4.538)

Model 1:no covariates were adjusted.

Model 2: adjusted for age, gender and race.

Model 3: adjusted for age, gender, race, education level, poverty-to-income ratio, alcohol intake, Hypertension, Diabetes, exercise routine, serum albumin, serum creatinine, low-density lipoprotein cholesterol, high-density lipoprotein cholesterol, triglyceride, urinary albumin and urinary creatinine.

**Fig. 4:**
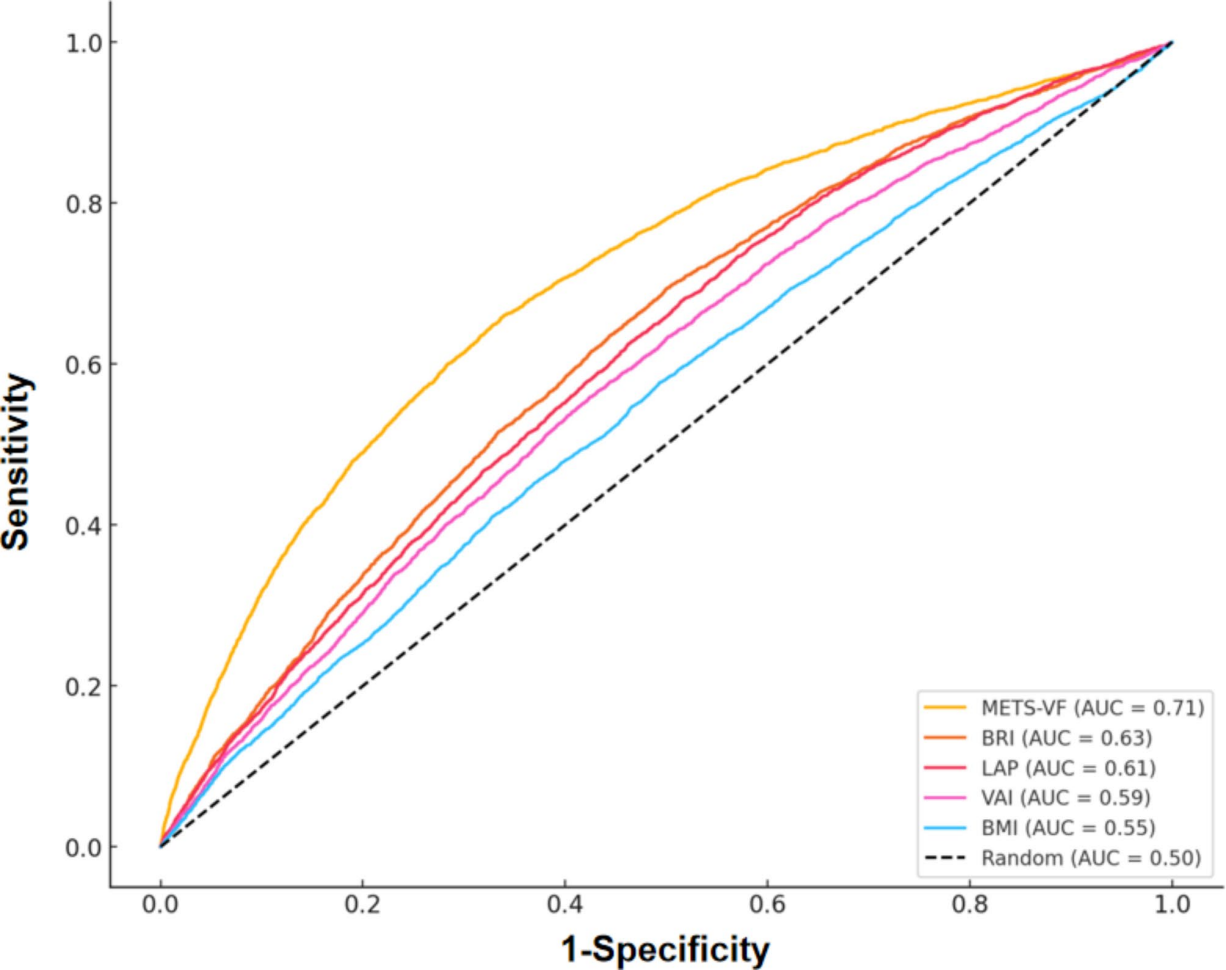
ROC curves for the prediction of CKD by adiposity indices. METS-VF, metabolic score for visceral fat; BRI, body roundness index; LAP, lipid accumulation product; VAI, visceral adiposity index; BMI, body mass index.

This study also assessed the diagnostic ability of abdominal obesity indices, including METS-VF, BRI, LAP, VAI, and BMI, by comparing their areas under the receiver operating characteristic (ROC) curve (AUC) values. The findings revealed that METS-VF had a notably higher AUC value (0.71) compared to BRI (0.63), LAP (0.61), VAI (0.59), and BMI (0.551) (Fig. 4).

## Discussion

This study represents the first cross-sectional analysis investigating the association between METS-VF and the prevalence of CKD in a nationally representative cohort of U.S. adults. Utilizing the extensive dataset from NHANES, the results demonstrate a significant nonlinear positive correlation between METS-VF and CKD prevalence. As the METS-VF score increases, the prevalence of CKD also rises, particularly at two critical thresholds—6.10 and 7.55—beyond which the risk of CKD increases substantially. These findings suggest that METS-VF could serve as an effective tool for identifying individuals at high risk for CKD.

The global incidence of CKD continues to rise, posing a significant public health challenge. Due to the often subtle nature of early CKD symptoms, many patients experience irreversible kidney damage before diagnosis. Consequently, early prevention of CKD is critical. Beyond genetic predispositions and conventional risk factors, obesity—particularly visceral obesity—has emerged as a major factor in the onset and progression of CKD. Visceral obesity is not only associated with metabolic disorders but also with an increased risk of kidney disease. It plays a critical role in the development of metabolic disorders and has been identified as a key contributor to the heightened risk of kidney disease^13,14^. Furthermore, excessive expansion of adipose tissue can lead to dysfunction and the abnormal accumulation of fat in ectopic locations, such as the kidneys, potentially contributing to renal impairment^4,15,16^.

BMI is widely used globally to assess obesity and estimate the risk of chronic kidney disease (CKD). However, BMI has several limitations, as it does not differentiate between fat and muscle mass, nor does it account for fat distribution, particularly across different age and gender groups. To address these limitations, several studies have evaluated the predictive ability of alternative obesity indices for CKD incidence, including LAP, BRI, and VAI^17–19^. The results indicate that these indices predict CKD more effectively than BMI, which is consistent with our results (see Fig. 4). In this study, METS-VF showed the highest area under the ROC curve for diagnosing CKD. Compared to LAP, VAI, and BRI, a 1 standard deviation increase in METS-VF was associated with a higher odds ratio (OR) for CKD, indicating the strongest correlation between METS-VF and abdominal obesity indices in CKD (Supplementary Table 1). These findings suggest that METS-VF is a valuable tool for predicting CKD incidence and identifying high-risk populations, potentially offering new opportunities for early intervention strategies.

In addition to these findings, it is also important to consider the potential impact of population differences when assessing the relationship between METS-VF and CKD. Although previous studies have explored the relationship between METS-VF and CKD, they have primarily focused on Asian populations, particularly participants recruited from the same city in China, with cohorts including individuals aged 40 and older, which may have certain limitations. In contrast, our study is based on the NHANES database, which includes a diverse U.S. population over a broader period (1999–2018), encompassing participants aged 20 and older from various geographical regions and racial groups. This broader population base and extended period not only enhance the generalizability of our findings but also increase their applicability across different populations and socioeconomic groups.

The calculation of METS-VF is influenced by demographic factors, including age, gender, WHtR, and METS-IR. This index incorporates individual characteristics, such as age and gender, enhancing its adaptability and accuracy in clinical practice, particularly across different population groups. Additionally, METS-VF integrates METS-IR, a key factor in evaluating obesity levels and cardiometabolic health^20,21^. Subgroup analysis further revealed that this positive correlation is more pronounced among males, likely due to hormonal differences that affect fat distribution and metabolism^22,23^. Males with visceral fat accumulation also exhibit reduced androgen synthesis, which can facilitate the redistribution of subcutaneous fat to the visceral area, exacerbating fat accumulation^24–26^.

The results also revealed that individuals engaging in regular physical activity exhibited a reduced risk of CKD, underscoring the importance of weight management. Numerous studies have demonstrated that even modest weight loss can significantly decrease the incidence of metabolic conditions, including CKD and diabetes. These findings underscore the critical need for targeted interventions focused on weight control, particularly among populations at high risk for CKD associated with METS-VF.

This study also identified two critical inflection points. When the METS-VF score was below 6.10, the risk of CKD was relatively low, suggesting that visceral fat accumulation at this stage may not yet pose a significant threat to kidney function. However, once the score exceeded 6.10, the risk of CKD increased markedly, suggesting that the body’s compensatory mechanisms had been overwhelmed, leading to kidney damage. Notably, when the METS-VF score surpassed 7.55, the risk of CKD increased substantially. This phenomenon is likely attributable to the synergistic effects of metabolic syndrome-related complications, including insulin resistance, the release of inflammatory cytokines, and elevated blood pressure^27,28^.

### Study strengths and limitations

This research possesses several notable strengths. Primarily, it is the inaugural study to employ the NHANES database for the examination of this relationship, thereby guaranteeing a comprehensive and representative sample. The large sample size provides robust statistical power, enabling us to identify two important inflection points. Second, the study design and rigorous statistical methods ensure the reliability and clinical relevance of the findings. METS-VF has demonstrated strong diagnostic efficacy for CKD risk prediction, which could inform future clinical applications.

Furthermore, this study is only a cross-sectional study which lacks sufficient evidence to test and infer mechanistic hypotheses. This research only assesses the correlation between METS-VF and CKD, but cannot determine the temporal relationship between the two. Future longitudinal studies would provide more comprehensive insights into the causal dynamics between METS-VF and CKD. Secondly, It is not feasible to account for all potential confounding factors in the study, which may leave the results vulnerable to some degree of interference. Introducing sensitivity analyses or stratified analyses in future research could further enhance the robustness of the findings. Additionally, while the NHANES database is somewhat representative, there may still be selection bias.

## Conclusion

This research, utilizing data from the NHANES database, established a notable association between elevated METS-VF levels and a heightened risk of CKD, reinforcing its potential as a predictor for CKD. The risk of CKD was found to rise sharply at critical thresholds of 6.10 and 7.55. It is advisable to lower METS-VF levels through dietary adjustments and physical activity in order to achieve an optimal range that may mitigate the risk of CKD.

## Electronic supplementary material

Below is the link to the electronic supplementary material.

Supplementary Material 1

## Data Availability

This study used publicly available data from the NHANES database (https://www.cdc.gov/nchs/nhanes/index.htm).

## Abbreviations

AUC: Area under the curve
BIA: Bioelectrical impedance analysis
BMI: Body mass index
CDC: Centers for Disease Control and Prevention
CKD: Chronic kidney disease
eGFR: Estimated glomerular filtration rate
FPG: Fasting plasma glucose
HDL-C: High density lipoprotein cholesterol
HC: Hip circumference
LDL-C: Low density lipoprotein cholesterol
METS-IR: Metabolic score for insulin resistance
METS-VF: Metabolism Score For Visceral Fat
NCHS: National Center for Health Statistics
NHANES: National Health and Nutrition Examination Survey
OR: Odds Ratio
PIR: Poverty income ratio
RCS: Restricted cubic spline
SD: Standard deviation
TG: Triglycerides
UACR: Urinary albumin/creatinine ratio
VAT: Visceral adipose tissue
WC: Waist circumference
WHtR: Waist-to-height ratio
WHR: Waist-to-hip ratio
95%CI: 95%Confidence Interval
