# Supplementary material for: Investigating the role of the metabolic score for visceral Fat in assessing the prevalence of chronic kidney disease from the NHANES 1999–2018
Source: Sci Rep. 2025 Jan 18;15:2397. doi: 10.1038/s41598-025-86723-3 (PMC11742988; doi:10.1038/s41598-025-86723-3)
Supplement: Supplementary file 1 — Supplementary Material 1 [file 41598_2025_86723_MOESM1_ESM.docx]

### Supplementary Table 1

| OR^1^(95%CI^2^) |  |  |  |
| --- | --- | --- | --- |
|  | Model 1 | Model 2 | Model 3 |
| VAI | 1.037 (1.030, 1.044) | 1.034 (1.026, 1.041) | 1.043 (0.998, 1.091) |
| LAP | 1.004 (1.004, 1.005) | 1.003 (1.002, 1.004) | 1.006 (1.002, 1.009) |
| BRI | 1.180 (1.164, 1.196) | 1.292 (1.236, 1.350) | 1.469 (1.362, 1.585) |

Model 1: no covariate were adjusted

Model 2: adjusted for age, gender and race

Model 3: adjusted for age, gender, race, education level, poverty-to-income ratio, alcohol intake, Hypertension, Diabetes, exercise routine, serum albumin , serum creatinine, low-density lipoprotein cholesterol, high-density lipoprotein cholesterol, triglyceride, urinary albumin and urinary creatinine.
